# Construction and integration of genetic linkage maps from three multi-parent advanced generation inter-cross populations in rice

**DOI:** 10.1186/s12284-020-0373-z

**Published:** 2020-02-14

**Authors:** Pingping Qu, Jinhui Shi, Tianxiao Chen, Kai Chen, Congcong Shen, Jiankang Wang, Xiangqian Zhao, Guoyou Ye, Jianlong Xu, Luyan Zhang

**Affiliations:** 1 The National Key Facility for Crop Gene Resources and Genetic Improvement, Institute of Crop Sciences, Chinese Academy of Agricultural Sciences, Beijing, 100081 China; 2 Agricultural Genomics Institute, Chinese Academy of Agricultural Sciences, Shenzhen, 518210 China; 3 Institute of Crop Science and Nuclear Technology Utilization, Zhejiang Academy of Agricultural Science, Hangzhou, 310021 China; 4 Genetics and Biotechnology Division, International Rice Research Institute, Baños, Laguna Philippines

**Keywords:** Multi-parent population, Quantitative trait locus (QTL), Linkage map, QTL mapping, Integrated map

## Abstract

**Background:**

The construction of genetic maps based on molecular markers is a crucial step in rice genetic and genomic studies. Pure lines derived from multiple parents provide more abundant genetic variation than those from bi-parent populations. Two four-parent pure-line populations (4PL1 and 4PL2) and one eight-parent pure-line population (8PL) were developed from eight homozygous *indica* varieties of rice by the International Rice Research Institute (IRRI). To the best of our knowledge, there have been no reports on linkage map construction and their integration in multi-parent populations of rice.

**Results:**

We constructed linkage maps for the three multi-parent populations and conducted quantitative trait locus (QTL) mapping for heading date (HD) and plant height (PH) based on the three maps by inclusive composite interval mapping (ICIM). An integrated map was built from the three individual maps and used for QTL projection and meta-analysis. QTL mapping of the three populations was also conducted based on the integrated map, and the mapping results were compared with those from meta-analysis. The three linkage maps developed for 8PL, 4PL1 and 4PL2 had 5905, 4354 and 5464 bins and were 1290.16, 1720.01 and 1560.30 cM in length, respectively. The integrated map was 3022.08 cM in length and contained 10,033 bins. Based on the three linkage maps, 3, 7 and 9 QTLs were detected for HD while 6, 9 and 10 QTLs were detected for PH in 8PL, 4PL1 and 4PL2, respectively. In contrast, 19 and 25 QTLs were identified for HD and PH by meta-analysis using the integrated map, respectively. Based on the integrated map, 5, 9, and 10 QTLs were detected for HD while 3, 10, and 12 QTLs were detected for PH in 8PL, 4PL1 and 4PL2, respectively. Eleven of these 49 QTLs coincided with those from the meta-analysis.

**Conclusions:**

In this study, we reported the first rice linkage map constructed from one eight-parent recombinant inbred line (RIL) population and the first integrated map from three multi-parent populations, which provide essential information for QTL linkage mapping, meta-analysis, and map-based cloning in rice genetics and breeding.

## Background

Rice is an important staple-food crop for nearly half of the world’s population. According to the International Grain Council (IGC), global rice production is projected to reach 505 million tons in 2019–2020, approximately 90% of which will be produced and consumed by Asians (Kong et al. 2015; Bazrkar-Khatibani et al. 2019). In the last several decades, the rice yield has increased slowly in most production countries, and breaking the barrier to yield growth has become a difficult challenge for breeders (Peng et al. 2004; Meng et al. 2016). It is promising to break through the yield stagnation by integrating conventional breeding methods with molecular marker technology and genomics (Liang et al. 2016).

Construction of linkage map by using molecular markers is a crucial step in genetic and genomic analysis that facilitates the discovery of quantitative trait locus/loci (QTLs) and provides chromosomal information for gene cloning and marker-assisted breeding (Meng et al. 2015; Palumbo et al. 2019). Since the publication of the first rice linkage map, which included 135 restriction fragment length polymorphism (RFLP) markers (McCouch et al. 1988), great progresses have been made in rice linkage map construction, especially with the development of high-throughput sequencing technology in recent years. Researchers have constructed a large quantity of maps in rice by using different types of populations and various molecular markers. For example, Harushima et al. (1998) reported a genetic linkage map containing 2275 cDNA markers covering 1521.6 cM in an F_2_ population of Nipponbare × Kasalath. Yin et al. (2015) constructed a linkage map containing 143 SSR markers in one *japonica* × *indica* genetic population consisting of 215 recombinant inbred lines (RILs). De Leon et al. (2016) constructed a high-density GBS-derived SNP linkage map with 2817 bins from a rice RIL population. Swamy et al. (2018) published 6 K SNP chip-derived SNP linkage maps in two doubled-haploid (DH) populations of rice. Kim (2018) developed a SNP map with 1954 bins for an F_2_ population.

Low marker number and density, and large number of co-localized markers are limitations of linkage maps constructed from single mapping populations, whereas consensus maps combining genetic information from multiple populations provide better genome coverage with higher marker density (Galeano et al. 2011). A consensus map can also validate marker order, identify chromosomal rearrangements and confirm the position of common markers across mapping populations (Salvi et al. 2009; Maccaferri et al. 2015). Furthermore, comparisons of QTLs on different linkage maps are based on the consensus map (Wen et al. 2017). Some tools for consensus map construction have been developed and widely used, such as JoinMap (Van Ooijen 2006), MergeMap (Wu et al. 2010), BioMercator (Arcade et al. 2004), LPmerge (Endelman and Plomion, 2014) and MultiPoint (Ronin et al. 2012). Using these tools, some integrated maps from bi-parent populations have been reported in rice. Swamy et al. (2011) integrated 15 individual maps, resulting in a consensus map with 531 markers and a total map length of 1821 cM. Swamy and Sarla (2011) constructed a consensus map from 11 maps with 699 markers and a map length of 1676 cM. Wu et al. (2016) developed a consensus map from 6 maps with 6970 markers and a total length of 1823.1 cM. Lei et al. (2018) constructed consensus maps from 6 maps with 7156 markers and a length of 1112.71 cM. Islam et al. (2019) developed a high-density consensus map with 12,327 simple sequence repeat (SSR) and SNP markers and a length of 2936 cM.

In the past 15 years, multi-parent advanced generation inter-cross (MAGIC) populations have become increasingly popular and have been developed in various plant species (Cavanagh et al. 2008; Kover et al. 2009; Huang et al. 2012; Mackay et al. 2014; Dell’Acqua et al. 2015; Pascual et al. 2015; Sannemann et al. 2015). Some MAGIC populations have been reported in rice. For example, International Rice Research Institute (IRRI) developed four MAGIC populations in rice (Bandillo et al. 2013), and Ogawa et al. (2018) developed a MAGIC population from eight high-yielding rice cultivars, and GWAS-based QTL mapping studies have also been conducted in those populations (Shen et al. 2017; Ogawa et al. 2018). Compared with bi-parent populations, MAGIC populations contain multiple alleles and possess an amplified number of recombination events and greater genetic diversity. However, the larger numbers of alleles and marker types at each locus in MAGIC populations complicate recombination frequency estimation and linkage map construction. For most MAGIC populations, genome-wide association studies (GWAS) have been employed for gene detection without linkage maps. However, GWAS have some disadvantages, such as lack of background control, and it doesn’t take advantage of linkage information. Accurate linkage analysis and linkage QTL mapping are essential for MAGIC populations. Several R packages and software have been developed to provide functions for constructing linkage maps and QTL mapping in MAGIC populations, including R/qtl (Broman et al. 2003), R/happy (Mott et al. 2000), R/mpMap (Huang and George 2011), and GAPL (integrated genetic analysis software for multi-parent pure-line populations) (Zhang et al. 2017; Zhang et al. 2019). Among these packages, GAPL is a stand-alone package used to build accurate linkage maps in a short time for MAGIC populations (Zhang et al. 2019). Inclusive composite interval mapping (ICIM) implemented in GAPL is applicable for QTL mapping in both four-parent and eight-parent pure-line populations, with high detection power, a low false discovery rate and accurate estimation of QTL positions and effects (Shi et al. 2019).

To further facilitate QTL discovery and variety development in rice, IRRI developed three rice MAGIC populations from eight diverse *indica* rice varieties (Meng et al. 2016), i.e., two four-parent RIL populations and one eight-parent RIL population. Meng et al. (2016, 2017) and Shen et al. (2017) conducted GWAS of abiotic tolerance, heading date (HD) and plant height (PH) in these populations. In this study, we focused on construction of linkage maps and integrated map, and linkage QTL mapping for the three multi-parent populations. Our objectives were (1) to construct individual linkage maps for the three multi-parent populations; (2) to construct an integrated map from the three individual maps; and (3) to conduct QTL mapping of HD and PH using the integrated map and compare the mapping results with those from individual populations with meta-analysis.

## Materials and Methods

### Plant Materials and Experimental Design

**Fig. 1:**
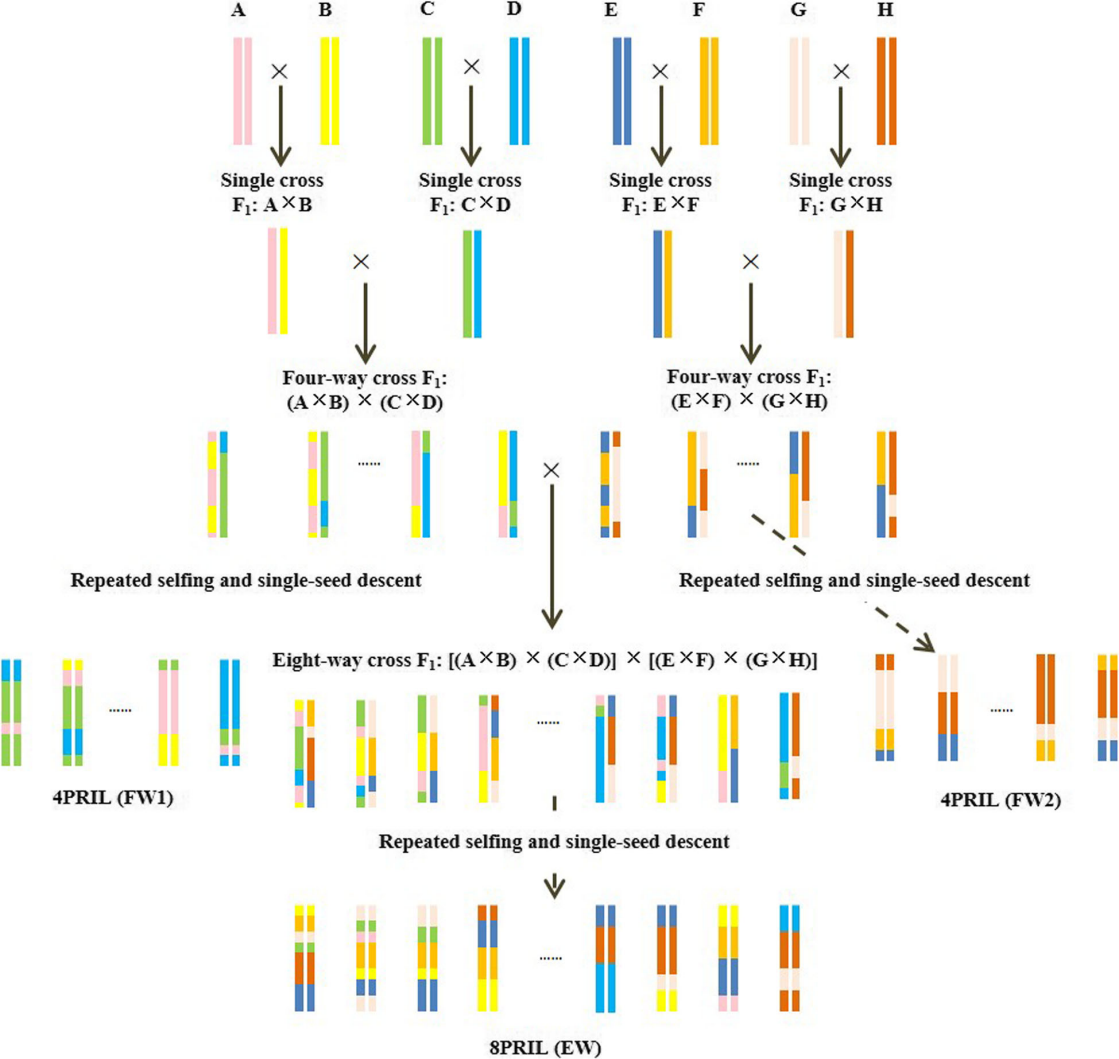
Development of three multi-parent pure-line populations in rice

Eight elite *indica* parents, namely, SAGC-08 (denoted A), HHZ5-SAL9-Y3-Y1 (B), BP1976B-2-3-7-TB-1-1 (C), PR33282-B-8-1-1-1-1-1 (D), FFZ1 (E), CT16658–5-2-2SR-2-3-6MP (F), IR68 (G) and IR02A127 (H), have been selected with genetic diversity to develop the three multi-parent RIL populations at the IRRI since 2008 (Meng et al. 2016). Four single crosses were made from the eight homozygous parents, denoted (A × B), (C × D), (E × F) and (G × H). Two four-way crosses were then generated from the four single crosses F_1_ plants, denoted (A × B) × (C × D) and (E × F) × (G × H), and advanced separately by single-seed descent (SSD) to produce two four-parent RIL populations (denoted 4PL1 and 4PL2). An eight-way cross was made by intercrossing 100 F_1_ plants of the four-way cross ABCD and 100 F_1_ plants of the four-way cross EFGH, denoted [(A × B) × (C × D)] × [(E × F) × (G × H)] (Fig. 1). The eight-parent RIL population was produced by SSD from 1000 eight-way cross F_1_ plants (denoted 8PL).

Field trials were conducted at the Pingxiang Experimental Station (113.85 °N, 27.6 °E) in Jiangxi Province (Shen et al. 2017). Seeds were sown in a seedling nursery on May 30, 2016. Seedlings were transplanted into the field 25 days after sowing. The three RIL populations, including 392 lines of 4PL1, 386 lines of 4PL2 and 566 lines of 8PL were planted in a randomized complete block design with two replications, with 10 plants in a row for each replication. The plant spacing was 17 cm between rows and 20 cm within each row. HD was recorded as the number of days from sowing to the date when 50% of the plants headed, whereas PH was measured based on five representative plants after complete heading. Mean values from the two replications were used for statistical analysis and QTL mapping.

### DNA Extraction and Genotyping

Lines from the three MAGIC populations were first genotyped and reported by Meng et al. (2017). The eight parents were re-genotyped in this study to obtain more accurate genotypic data and reduce the rate of missing data. Genomic DNA for SNP genotyping was isolated from approximately 100-mg fresh leaf samples of 5-week-old seedlings using a modified cetyltrimethylammonium bromide (CTAB) method (Murray and Thompson, 1980). Genotyping was performed using a customized rice 55 K SNP array containing 56,897 SNP screened from the 3 K Rice Genome Project (Project 2014; Meng et al. 2017; Wang et al. 2018). Target DNA preparation, chip hybridization, and array processing were conducted by CapitalBio Technology (Beijing, China) according to the Affymetrix Axiom® 2.0 assay protocol. Finally, 39,070 high-quality (poly high-resolution) SNPs with genetic diversity were selected based on comparison to the *Oryza sativa* cv. Nipponbare IRGSP 1.0 reference genome (Kawahara et al. 2013). For convenience, SNPs were renamed based on their physical locations.

### Construction of Individual and Integrated Maps

Quality control of the genotypic data was performed for the three populations separately using GAPL V1.2 software (Zhang et al. 2019). First, using the SNP (SNP genotypic data conversion) functionality in GAPL, DNA base data were converted into a format that could be imported into the software. SNPs showing non-polymorphism in parents or progenies or that were missing in one or more parents were filtered out. Second, using the BIN (binning of redundant markers) function, markers with more than 10% missing values were deleted, meanwhile, the marker with the minimum percentage of missing data was selected to represent the co-localized markers. A set of co-localized markers in a specific population was defined as one bin. Finally, markers having more than 3% heterozygosity in progenies and progenies having more than 5% heterozygosity were removed. SNPs retained after filtering were used for map construction.

Linkage maps of 8PL, 4PL1 and 4PL2 were constructed separately using the PLM (map construction in multi-parent derived pure-line populations) function in GAPL. First, SNPs with a known chromosome ID on a physical map were anchored. The unanchored SNP markers were grouped based on a minimum logarithm of odds (LOD) score of 3. Second, the nearest-neighbour algorithm and two-opt algorithm from the travelling salesman problem (TSP, Lin and Kernighan, 1973) were adopted for marker ordering. Finally, the sum of adjacent recombination frequencies calculated with a five-marker window size was used as the rippling criterion. Recombination frequency was converted into map distance by the Kosambi mapping function.

The integrated map was built by using MergeMap V4.1 software (Wu et al. 2010) based on the individual maps from 8PL, 4PL1 and 4PL2. Equal weight was given to the three individual maps. The R package *LinkageMapView* was used for visualization of the constructed maps (Ouellette et al. 2017).

### QTL Analysis of the Three Populations

QTL mapping of HD and PH was conducted separately in 8PL, 4PL1 and 4PL2 by using ICIM with the PLQ (QTL or gene detection in multi-parent derived pure-line populations) function in GAPL. The scanning step was set at 0.1 cM. Probabilities of adding and removing variables in stepwise regression were set at 0.001 and 0.002, respectively. The LOD threshold was determined by a permutation test with 1000 runs, and the type-I error was set to 0.05. Mapping results from 8PL were compared with the results obtained by GWAS in Shen et al. (2017), where 0.00001 was used as the threshold for the *P*-value.

### QTL Projection and Meta-QTL Analysis

BioMercator V4.2 was used to project the detected HD and PH QTLs onto the integrated map and perform QTL meta-analysis (Veyrieras et al. 2007, Sosnowski et al. 2012). Based on the estimated position, LOD score, phenotypic variation explained (PVE) by each QTL, and confidence interval, these QTLs were projected onto the integrated map. Meta-analysis of the projected QTL clusters was performed using Goffinet’s and Gerber’s algorithm (Goffinet and Gerber 2000) for each chromosome. The lowest Akaike information criterion (AIC) value was used to select the best QTL model. The position and 95% confidence interval of each meta-QTL (MQTL) were calculated. QTL mapping of DH and PH using the PLQ function in GAPL was also conducted with the integrated map. The mapping parameters were the same as those used for QTL mapping with individual maps.

## Results

### General Information on the Genotypic and Phenotypic Data

A total of 39,070 markers were used to filter the pipeline in the three MAGIC populations. The missing rate of these markers is shown in Additional file 8: Figure 1A. In 8PL, 4PL1 and 4PL2, there were 279, 252 and 270 markers whose missing rates were greater than 10%; percent of 13.17, 12.03, and 4.48 of the markers had a heterozygosity level over 3%, respectively (Additional file 8: Figure 1B). After removing these markers as well as SNPs with non-polymorphism in parents or progenies or that were missing in one or more parents, 13,254, 10,825 and 13,479 SNPs were retained for 8PL, 4PL1 and 4PL2, respectively. A total of 518, 364 and 356 progenies were kept for 8PL, 4PL1 and 4PL2, respectively, after controlling for progeny heterozygosity. After removing co-localized markers, 5906, 4368 and 5481 markers were retained in the 8PL, 4PL1 and 4PL2 populations and then used for linkage map construction, respectively. Populations of 8PL and 4PL1 had 2352 common markers; populations of 8PL and 4PL2 had 2147 common markers; populations of 4PL1 and 4PL2 had 1379 common markers; and the three populations had 942 markers in common (Additional file 8: Figure 1C).

The phenotypic variation in the eight parents and the three RILs populations are shown in Additional files 1 and 2: Tables S1 and S2, respectively. The range of variations in progenies was greater than that in parents, reflecting the presence of transgressive segregation. The kurtosis and skewness of HD and PH were close to zero, except for those of PH in 4PL2. The broad-sense hereditability (H^2^) was 0.9867, 0.9346 and 0.9736 for HD and 0.7766, 0.7711 and 0.9217 for PH in 8PL, 4PL1 and 4PL2, respectively.

### Individual and Integrated Maps

**Fig. 2:**
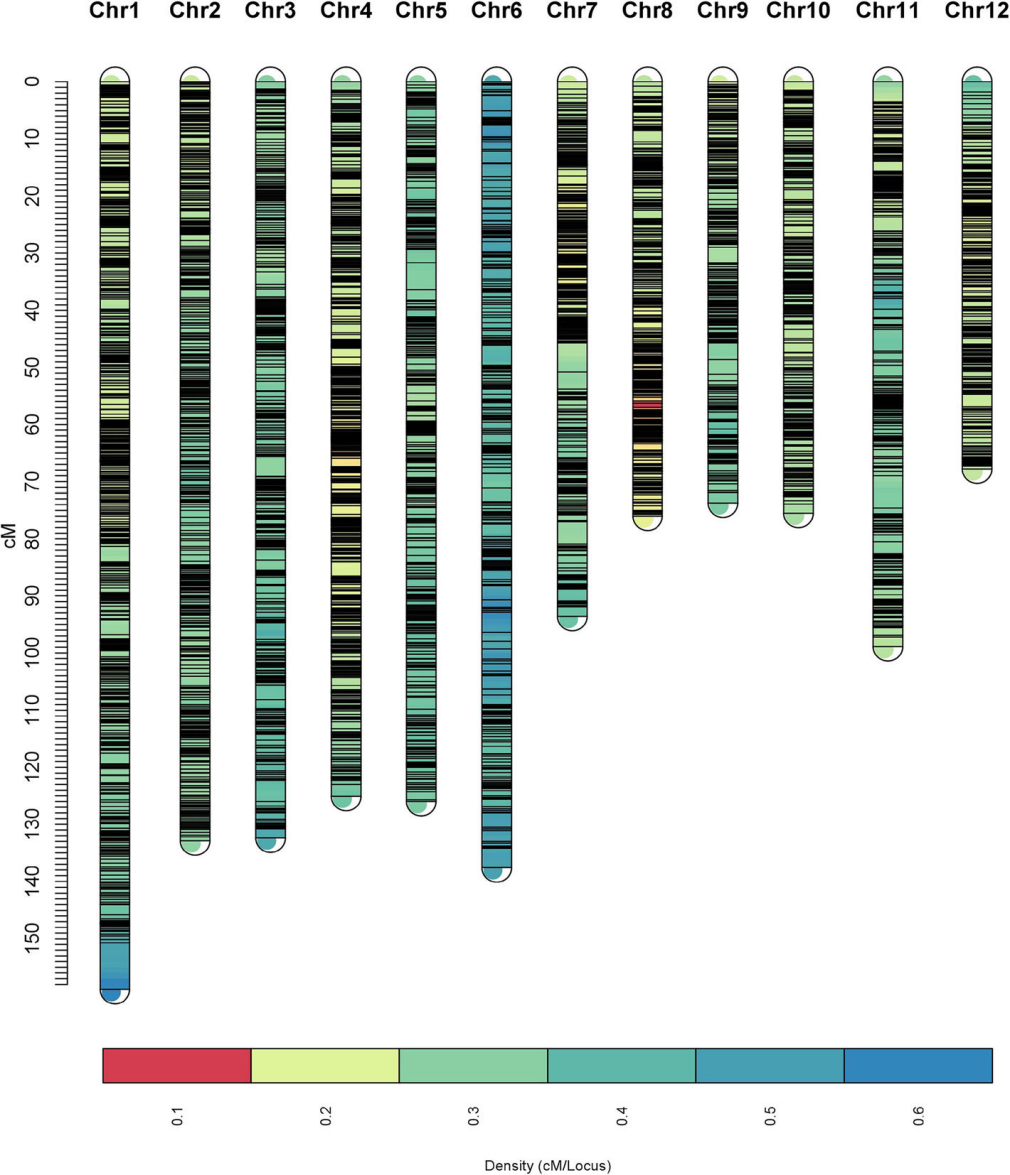
Linkage map constructed by GAPL from 8PL

**Fig. 3:**
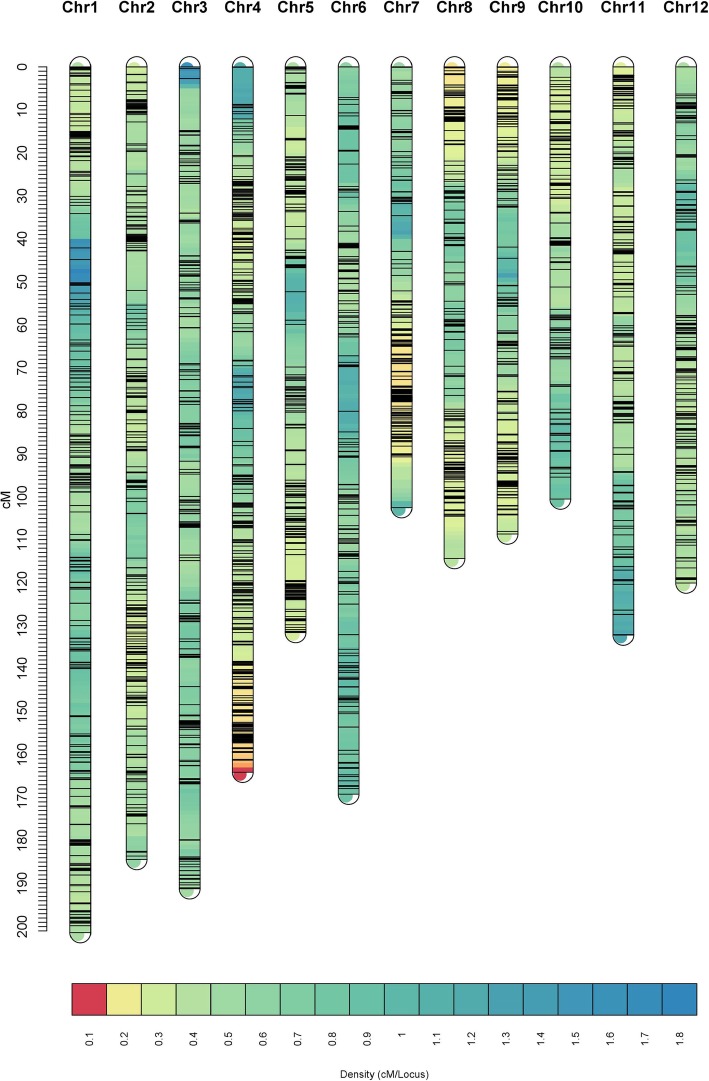
Linkage map constructed by GAPL from 4PL1

**Fig. 4:**
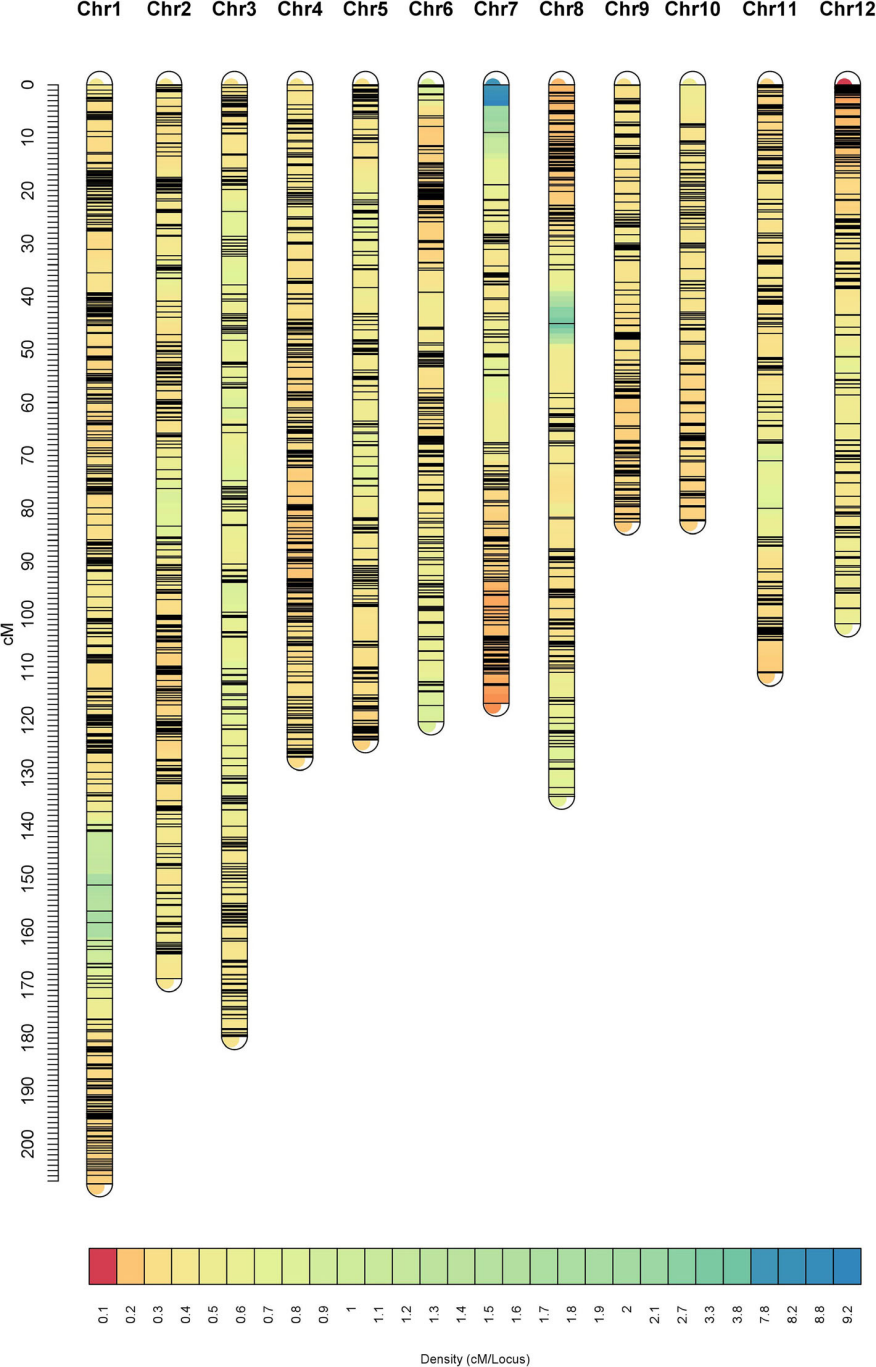
Linkage map constructed by GAPL from 4PL2

The distribution of SNPs and linkage map information for the three populations are shown in Table 1. For 8PL, the full genome was 1290.16 cM in length and included 5906 markers across the 12 chromosomes (Fig. 2). The number of bins was 5905, i.e., 5905 markers had unique map positions. The average distance between adjacent bins was 0.22 cM. The most saturated chromosome was chromosome 8, which had an average bin distance of 0.15 cM, while chromosome 6 had the largest average bin distance of 0.39 cM. The average chromosome length was 107.51 cM, with the longest being chromosome 1 (150.66 cM) and the shortest being chromosome 12 (67.79 cM) (Table 1). For 4PL1, the entire genome spanned 1720.01 cM and contained 4368 markers (Fig. 3). The number of bins was 4354. The average bin distance was 0.40 cM, and the average chromosome length was 143.35 cM. Chromosome 7 had the smallest average bin distance of 0.28 cM, whereas chromosome 6 had the largest average bin distance of 0.66 cM. Chromosome 1 was the longest, i.e., 201.39 cM in length, and chromosome 10 was the shortest, i.e., 100.54 cM in length. For 4PL2, the entire genome was 1560.30 cM in length and contained 5481 markers (Fig. 4). The number of bins was 5464. The average bin distance was 0.29 cM, with the smallest average bin distance of 0.22 cM on chromosome 4, whereas the largest average bin distance of 0.44 cM on chromosome 3. The average chromosome length was 130.03 cM, with the longest being chromosome 1 (207.53 cM) and the shortest being chromosome 10 (82.39 cM). Markers were approximately evenly distributed among the three individual maps from the three populations. The longest bin intervals were 5.64, 12.98, and 13.14 cM for 8PL, 4PL1 and 4PL2, respectively, whereas the shortest bin intervals were all equal to 0.1 cM. The 8PL map had the largest marker number, shortest genome length, and shortest average bin distance, whereas the 4PL1 map had the smallest marker number, longest genome length, and longest average bin distance (Table 1, Figs. 2, 3 and 4).

**Table 1:** Information on individual linkage maps for the three multi-parent RIL populations

Chr.	8PL						4PL1					4PL2			
	No. of markers	No. of bins	Length (cM)	Max distance (cM)	Average distance (cM)^*a*^	No. of markers	No. of bins	Length (cM)	Max distance (cM)	Average distance (cM)	No. of markers	No. of bins	Length (cM)	Max distance (cM)	Average distance (cM)
1	764	764	150.66	2.70	0.20	395	393	201.39	10.96	0.51	731	729	207.53	10.05	0.28
2	589	589	132.88	1.63	0.23	486	484	184.42	10.43	0.38	577	576	168.84	7.07	0.29
3	490	490	132.36	3.68	0.27	357	357	191.19	12.98	0.54	409	408	179.74	9.11	0.44
4	735	735	125.14	2.49	0.17	550	549	164.14	8.63	0.30	581	579	126.97	3.45	0.22
5	466	465	125.99	4.75	0.27	376	374	131.64	9.88	0.35	385	384	123.7	6.60	0.32
6	355	355	137.56	3.49	0.39	259	257	169.15	10.37	0.66	402	402	120.27	6.63	0.30
7	481	481	93.57	5.19	0.19	365	364	102.23	11.76	0.28	460	458	121.00	12.65	0.26
8	493	493	76.04	1.94	0.15	373	373	114.40	9.94	0.31	459	455	134.39	13.14	0.30
9	329	329	73.69	2.90	0.22	345	343	108.68	9.23	0.32	353	352	82.61	4.10	0.24
10	388	388	75.55	1.69	0.19	252	250	100.54	6.06	0.40	320	320	82.39	7.37	0.26
11	429	429	98.93	5.64	0.23	343	343	132.17	6.01	0.39	405	403	111.07	9.02	0.28
12	387	387	67.79	2.21	0.18	267	267	120.06	8.27	0.45	399	398	101.79	5.40	0.26
Total	5906	5905	1290.16	5.64	0.22	4368	4354	1720.01	12.98	0.40	5481	5464	1560.30	13.14	0.29

^*a*^Average distance between two adjacent bins in cM

Contribution of each parent can be quantified by the proportion of the parental genome in the progeny (Wang and Bernardo 2000). In MAGIC population, it cannot be directly estimated by the bi-allelic SNP markers. However, after the map construction in GAPL, any missing and incomplete marker (e.g. bi-allelic SNP marker) can be imputed as complete marker, i.e., locus with eight alleles. Therefore, contribution of each parent can be calculated after imputation. In 4PL1, the proportion was 0.3183, 0.1184, 0.2683 and 0.2950 for parents A to D, respectively. In 4PL2, the proportion was 0.3536, 0.1725, 0.2318 and 0.2421 for parents E to H, respectively. In 8PL, the proportion was 0.1372, 0.1043, 0.1314, 0.1198, 0.1901, 0.0696, 0.1236 and 0.1240 for parents A to H, respectively. Theoretically, each parent contributed equally to MAGIC populations. The variation on parental contribution observed may be caused by genetic drift, potential selection, marker ascertainment bias and sampling errors.

**Fig. 5:**
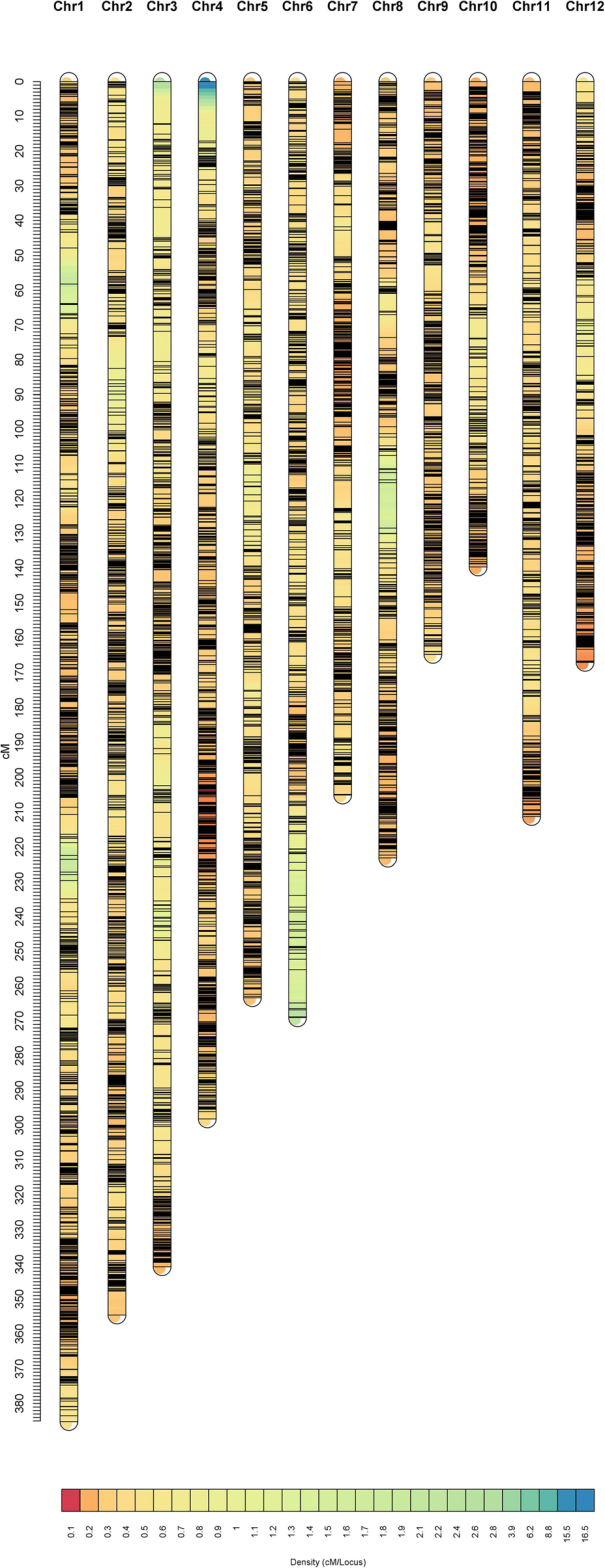
Integrated map constructed from the three individual linkage maps for 8PL, 4PL1 and 4PL2

The integrated map based on the three individual maps comprised 10,785 markers, 10,033 of which had unique map positions (Fig. 5). The genome was 3022 cM in length, with an average distance of 0.30 cM between adjacent bins (Table 2). Chromosome 1 had the largest number of 1403 SNPs, and chromosome 10 contained the smallest number of 639 SNPs. The average chromosome length was 251.84 cM. Chromosome 1 was the longest chromosome with total length of 385.18 cM and a mean distance of 0.29 cM between adjacent bins. Chromosome 10 was the shortest with total length of 139.54 cM and a mean bin distance of 0.24 cM. Chromosome 10 had the smallest average bin size of 0.24 cM, whereas chromosome 6 had the largest average bin size of 0.41 cM. The integrated map had no gaps longer than 17.05 cM. The average bin distance in the integrated map was larger than that in 8PL but smaller than that in 4PL1 and 4PL2. The genome length of the integrated map was greater than the three individual maps. Co-localized markers in some individual maps could be separated in other maps. Those markers will also be separated in the integrated map, leading to a longer genome in integrated map.

**Table 2:** Information on the integrated map from the three individual maps

Chr.	No. of markers	No. of bins	Length (cM)	Max distance (cM)	Average distance between bins (cM)	Average distance between markers (cM)
1	1403	1325	385.18	10.48	0.29	0.27
2	1152	1092	354.61	9.14	0.32	0.31
3	892	840	340.71	12.10	0.41	0.38
4	1236	1163	298.23	17.05	0.26	0.24
5	826	782	263.41	6.53	0.34	0.32
6	741	661	269.15	9.62	0.41	0.36
7	854	786	205.13	8.00	0.26	0.24
8	857	784	223.17	13.15	0.28	0.26
9	706	652	164.67	7.54	0.25	0.23
10	639	573	139.54	5.84	0.24	0.22
11	765	698	211.3	5.38	0.30	0.28
12	714	677	166.98	5.39	0.25	0.23
Total	10,785	10,033	3022.08	17.05	0.30	0.28

### QTL Analysis Based on the Three Linkage Maps

From permutation tests, LOD thresholds at the 0.05 significance level were calculated at 8.59, 5.22 and 5.40 for 8PL, 4PL1 and 4PL2, respectively. The estimated positions and effects of the detected QTLs are given in Table 3 for 8PL and Additional files 3 and 4: Tables S3 and S4 for 4PL1 and 4PL2, respectively.

**Table 3:** QTLs for heading date and plant height in the eight-way-cross RIL population

QTL	Chr.	Pos. (CI)^*a*^ (cM)	Left marker	Right marker	LOD	PVE (%)^*b*^	Genotypic effect								Cloned gene
							*a*_1_	*a*_2_	*a*_3_	*a*_4_	*a*_5_	*a*_6_	*a*_7_	*a*_8_	
*qHD6.1*	6	11.10 (10.95–11.15)	Chr6–1,826,585	Chr6–2,336,493	9.42	5.15	−1.50	−5.38	− 5.72	−3.26	− 0.46	− 2.44	− 0.53	19.30	
*qHD6.2*	6	70.50 (70.15–70.85)	Chr6–3,305,201	Chr6–2,927,171	42.38	15.66	−2.54	−3.04	−0.44	1.52	−4.02	−1.30	9.43	0.39	*Hd3a*
*qHD8*	8	73.40 (73.25–73.55)	Chr8–4,701,356	Chr8–4,892,161	10.64	3.48	−1.00	2.36	3.50	−1.44	−1.46	−2.56	2.06	−1.45	*Ghd8*
*qPH1.1*	1	7.10 (7.05–7.15)	Chr1–38,264,136	Chr1–38,383,144	44.81	6.92	−4.12	−7.18	12.18	−3.40	−4.67	17.53	−5.99	−4.34	*sd1*
*qPH1.2*	1	11.80 (11.75–11.85)	Chr1–37,101,140	Chr1–37,595,895	36.93	5.92	−4.84	−4.20	11.54	−5.12	−5.06	16.52	−2.79	−6.04	*Psd1*
*qPH2*	2	129.50 (129.45–129.55)	Chr2–35,144,957	Chr2–26,805,011	15.37	2.13	1.99	3.60	−3.57	−3.60	−3.79	−1.51	1.96	4.92	
*qPH8*	8	21.00 (20.95–21.15)	Chr8–5,727,201	Chr8–6,128,189	8.74	1.42	−2.86	3.60	4.07	−2.94	−1.34	−0.77	2.04	−1.79	
*qPH11*	11	57.00 (56.85–57.05)	Chr11–22,884,317	Chr11–21,254,126	9.32	1.28	4.20	−1.84	0.44	−4.13	2.61	−3.45	2.67	−0.50	
*qPH12*	12	48.40 (48.25–48.55)	Chr12–21,784,888	Chr12–21,468,631	14.61	2.01	1.47	0.88	−1.30	−1.88	3.48	−3.05	4.82	−4.44	

^*a*^Position in cM and 1-LOD confidence interval (CI)

^*b*^Percentage of phenotypic variance explained

For HD, three QTLs were detected in 8PL, explaining a total of 24.29% of the phenotypic variance. Two were located on chromosome 6, and one, on chromosome 8. *qHD6.1*, located at 70.50 cM on chromosome 6, had the largest LOD score of 42.38 and the largest PVE of 15.66% (Table 3). At *qHD6.1*, parents A, B, C, E, and F provided alleles that reduced HD. Given that the length of the confidence interval was 0.5 Mb, i.e., the distance between a gene and an adjacent marker of the detected QTL was less than 0.5 Mb on the rice physical map, the gene and the QTL were declared to coincide. *qHD6.2* and *qHD8* coincided with *Hd3a* (*LOC_Os06g06320*), located from 2,940,004 to 2,942,452 bp (Taoka et al. 2011), and *Ghd8* (*LOC_Os08g07740*), from 4,334,739 to 4,333,846 bp (Yan et al. 2011), respectively. In 4PL1, seven HD QTLs were detected, explaining a total of 60.18% of the phenotypic variance. Two were located on chromosome 8, and one each, on chromosomes 3, 4, 6, 7 and 11 (Additional file 3: Table S3). *qHD6*, located at 60.9 cM on chromosome 6 and coinciding with *Hd3a*, had the largest LOD score of 51.86 and the largest PVE of 28.63%. In 4PL2, nine QTLs affecting HD were detected, explaining a total of 55.83% of the phenotypic variance, among which *qHD8* had the largest LOD score of 54.71 and largest PVE of 25.07% and was close to *Ghd8* (Additional file 4: Table S4). In addition, *qHD3.1*, located at 13.10 cM on chromosome 3, was close to the reported gene *HD6* (*LOC_Os03g55389*) that was located from 31,508,811 to 31,514,460 bp and responsible for a delay in HD in long day conditions (Yamamoto et al. 2000).

For PH, six QTLs were detected in 8PL, explaining a total of 19.68% of the phenotypic variance. Two were located on chromosome 1, and one each, on chromosomes 2, 8, 11 and 12 (Table 3). *qPH1.1* was located at 7.10 cM on chromosome 1, having the largest LOD score of 44.81 and the largest PVE of 6.92%. Alleles from parents A, B, D, E, G and H at *qPH1.1* reduced PH. The cloned dwarf gene *sd1* (*LOC_Os01g66100*), located from 38,381,423 to 38,384,165 bp (Monna et al. 2002), and the semi-dwarf gene *Psd1* (*LOC_Os01g60740*), located from 35,129,858 to 35,130,917 bp (Li et al. 2014), were close to *qPH1.1* and *qPH1.2*. Nine QTLs were detected in 4PL1, two each on chromosomes 5 and 8 and one each on chromosomes 1, 3, 6, 7 and 10, explaining a total of 74.99% of the phenotypic variance (Additional file 3: Table S3). *qPH1* was located at 181.7 cM on chromosome 1, near the cloned gene *sd1*, and had the largest LOD score of 98.95 and largest PVE of 55.80%. In 4PL2, 10 QTLs were detected, located on chromosomes 1, 2, 5, 7, 8, and 10 and explaining a total of 42.33% of the phenotypic variance (Additional file 4: Table S4). *qPH1.2,* with the largest LOD score of 164.76 and the largest PVE of 34.85, coincided with *d61*, located from 29,927,543 to 29,931,487 bp (Yamamuro et al. 2000).

### QTL Detection Based on Meta-Analysis

Based on the QTLs detected in 8PL, 4PL1 and 4PL2, a total of 44 MQTLs were identified on the integrated map, including 19 for HD and 25 for PH (Table 4, Additional file 9: Figure S2). Four of them were detected in two populations, i.e. *MqHD3.2*, *MqHD11.2* and *MqPH10.2* was detected in both 4PL1 and 4PL2; *MqPH8.2* was detected in both 4PL1 and 8PL. The others were detected in one population. For HD, *MqHD3.1* was located at 75.33 cM, with a confidence interval from 73.68 to 75.48 cM, and thus coincided with the cloned gene *Hd6*; *MqHD6.2* and *MqHD6.4* coincided with *Hd3a*; and *MqHD8.1* and *MqHD8.2* coincided with *Ghd8*. For PH, *MqPH1.6* and *MqPH1.7* coincided with *sd1*; *MqPH1.2* coincided with *d61*; and *MqPH1.5* coincided with *Psd1*.

**Table 4:** QTLs for heading date and plant height detected by meta-analysis

Meta-QTL	Chr.	Pos. (cM)	Start (cM)	End (cM)	QTL Belonging	Cloned gene
*MqHD1.1*	1	182.55	181.90	183.00	*4PL2_qHD1.2*	
*MqHD1.2*	1	206.72	206.67	206.87	*4PL2_qHD1.1*	
*MqHD2*	2	82.32	82.17	82.47	*4PL2_qHD2*	
*MqHD3.1*	3	75.33	73.68	75.48	*4PL2_qHD3.1*	*Hd6*
*MqHD3.2*	3	116.33	115.47	117.19	*4PL1_qHD3/4PL2_qHD3.2*	
*MqHD4*	4	51.03	50.98	51.78	*4PL1_qHD4*	
*MqHD6.1*	6	10.17	10.02	10.22	*8PL_qHD6.1*	
*MqHD6.2*	6	61.89	61.54	62.24	*8PL_qHD6.2*	*Hd3a*
*MqHD6.3*	6	80.54	80.49	80.79	*4PL2_qHD6*	
*MqHD6.4*	6	93.13	92.68	93.78	*4PL1_qHD6*	*Hd3a*
*MqHD7.1*	7	49.58	49.53	50.03	*4PL1_qHD7*	
*MqHD7.2*	7	109.68	109.43	110.23	*4PL2_qHD7*	
*MqHD8.1*	8	2.58	2.43	2.73	*8PL_qHD8*	*Ghd8*
*MqHD8.2*	8	13.02	12.67	13.07	*4PL2_qHD8*	*Ghd8*
*MqHD8.3*	8	42.52	42.27	43.27	*4PL1_qHD8.1*	
*MqHD8.4*	8	118.77	118.42	119.12	*4PL1_qHD8.2*	
*MqHD11.1*	11	94.69	94.44	95.64	*4PL1_qHD11*	
*MqHD11.2*	11	99.58	99.22	99.94	*4PL1_qHD11 / 4PL2_qHD11*	
*MqHD11.3*	11	102.34	101.59	102.49	*4PL2_qHD11*	
*MqPH1.1*	1	24.31	23.76	24.36	*4PL2_qPH1.3*	
*MqPH1.2*	1	32.87	32.62	32.92	*4PL2_qPH1.2*	*d61*
*MqPH1.3*	1	57.69	57.64	58.04	*4PL2_qPH1.1*	
*MqPH1.4*	1	107.05	106.80	107.30	*8PL_qPH1.3*	
*MqPH1.5*	1	197.40	197.35	197.45	*8PL_qPH1.2*	*Psd1*
*MqPH1.6*	1	198.75	198.30	199.40	*4PL1_qPH1*	*sd1*
*MqPH1.7*	1	200.60	200.55	200.65	*8PL_qPH1.1*	*sd1*
*MqPH2.1*	2	32.36	32.31	32.41	*8PL_qPH2*	
*MqPH2.2*	2	82.01	81.76	82.36	*4PL2_qPH2*	
*MqPH3*	3	118.00	117.15	118.15	*4PL1_qPH3*	
*MqPH5.1*	5	48.93	48.28	50.28	*4PL2_qPH5*	
*MqPH5.2*	5	84.64	83.79	85.29	*4PL1_qPH5.1*	
*MqPH5.3*	5	138.74	138.49	138.99	*4PL1_qPH5.2*	
*MqPH6*	6	143.22	141.87	143.37	*4PL1_qPH6*	
*MqPH7.1*	7	5.97	5.82	6.02	*4PL2_qPH7.2*	
*MqPH7.2*	7	43.87	43.42	44.02	*4PL2_qPH7.1*	
*MqPH7.3*	7	90.98	89.63	93.83	*4PL1_qPH7*	
*MqPH8.1*	8	12.42	11.77	13.17	*4PL2_qPH8*	
*MqPH8.2*	8	114.68	114.59	114.78	*4PL1_qPH8.1/8PL_qPH8*	
*MqPH8.3*	8	119.12	118.77	119.37	*4PL1_qPH8.1*	
*MqPH10.1*	10	5.95	5.00	6.50	*4PL2_qPH10.2*	
*MqPH10.2*	10	20.14	20.04	20.24	*4PL1_qPH10/4PL2_qPH10.1*	
*MqPH10.3*	10	31.82	31.07	32.37	*4PL1_qPH10*	
*MqPH11*	11	80.16	80.01	80.21	*8PL_qPH11*	
*MqPH12*	12	83.71	83.56	83.86	*8PL_qPH12*	

### QTL Analysis Based on the Integrated Map

From permutation tests, LOD thresholds at the 0.05 significance level were calculated at 9.08, 5.71 and 5.87 for 8PL, 4PL1 and 4PL2, respectively. The estimated positions and effects of the detected QTLs affecting HD and PH (denoted IQTLs) are given in Table 5 and Additional files 5 and 6 Tables S5 and S6. The LOD score profiles across the genome in the three populations are shown in Additional file 10: Figure S3. In 8PL, five QTLs were detected for HD, and three were detected for PH (Table 5). *IqHD6.2* was mapped to 92.50 cM with a confidence interval from 92.15 to 92.75 cM and had the largest LOD score of 46.04 and the largest PVE of 21.05%. Parents A to F provided the alleles from *IqHD6.2* that reduced HD. In 4PL1, nine QTLs were identified for HD, and ten were identified for PH. The PVE by a single QTL ranged from 1.46 to 24.88% for HD and from 0.66 to 16.73% for PH (Additional file 5: Table S5). *IqHD6* had the highest LOD score of 66.50 and largest PVE of 24.88%. Parents A, B and C provided the alleles from *IqHD6* that reduced HD. In the 4PL2 population, 10 QTLs were identified for HD, and 12, for PH. *IqPH1.2* had the highest LOD score of 119.95 and the highest PVE of 21.74%. Parents F and G provided the alleles from *IqPH1.2* that reduced PH.

**Table 5:** QTLs for heading date and plant height based on the integrated map in 8PL

QTL	Chr.	Pos. (CI) ^*a*^ (cM)	Left marker	Right marker	LOD	PVE (%) ^*b*^	Genotypic effect							
							*a*_1_	*a*_2_	*a*_3_	*a*_4_	*a*_5_	*a*_6_	*a*_7_	*a*_8_
*IqHD1*	1	263.71 (263.06–265.16)	Chr1–6,588,793	Chr1–5,958,935	9.97	3.54	2.61	2.47	1.19	−1.34	−1.37	−0.62	−1.37	−1.56
*IqHD2*	2	84.90 (83.15–85.75)	Chr2–3,433,320	Chr2–5,042,884	12.92	5.03	1.26	−2.19	0.73	−1.27	2.68	−1.33	2.18	−2.05
*IqHD6.1*	6	8.10 (7.75–8.45)	Chr6–5,681,741	Chr6–26,069,125	12.68	4.67	−1.44	−1.81	1.12	1.77	−1.64	−1.43	2.52	0.91
*IqHD6.2*	6	92.50 (92.15–92.75)	Chr6–3,305,201	Chr6–2,927,171	46.04	21.05	−0.81	−1.67	−1.21	−0.84	−2.64	−2.65	9.03	0.79
*IqHD8*	8	42.40 (42.35–42.55)	Chr8–6,181,174	Chr8–9,288,403	9.11	3.23	2.05	−0.41	1.53	−3.72	−0.82	− 0.11	−0.45	1.94
*IqPH1*	1	358.21 (358.06–358.26)	Chr1–38,503,485	Chr1–38,103,681	19.49	9.47	−2.49	− 2.86	7.78	−5.47	−4.61	7.18	3.34	−2.88
*IqPH2*	2	304.21 (304.16–304.76)	Chr2–418,968	Chr2–2,416,287	11.11	5.19	−4.67	1.96	3.66	−1.56	−2.36	4.85	0.86	−2.74
*IqPH11*	11	46.10 (44.65–46.95)	Chr11–19,718,068	Chr11–23,080,452	10.36	4.73	−3.06	−2.47	− 3.85	−4.11	2.79	3.99	2.57	4.15

^*a*^Position in cM and 1-LOD confidence interval (CI)

^*b*^Percentage of phenotypic variance explained

## Discussion

### Comparison of the Three Individual Maps with their Integrated Map

To the best of our knowledge, this study provided the first linkage map from an eight-parent RIL population in rice and the first integrated map constructed from a number of multi-parent populations in rice. Three linkage maps were first constructed from three multi-parent populations, and an integrated map was then built from the three individual maps. Among the three individual maps, the 8PL map had a larger number of bins, shorter genome length, and shorter average bin size than the 4PL1 and 4PL2 maps (Table 1, Figs. 2, 3 and 4). At one locus, the eight-parent population contained twice as many alleles as the four-parent population. The greater number of recombination events and higher genetic diversity in 8PL resulted in more unique locations and higher saturation of markers on linkage maps. The much larger number of markers contained in the integrated map than in the individual maps led to the longer genome of the integrated map. In addition, the integrated map had more bins and a longer length than the three individual maps, with a larger average bin size than that in 8PL but a smaller average bin size than those in 4PL1 and 4PL2. The marker order in the integrated map was not completely consistent with that in the individual maps.

For each linkage map, over ten thousand markers were selected after the first step of filtering, but only half of them were retained after binning. Bin number is much smaller than marker number in linkage mapping populations with limited crossovers, especially when high density markers are used for genotyping. For example, in an actual cowpea population consisting of 305 RILs from an eight-way cross reported by Huynh et al. (2018), 32,114 SNPs were distributed on 11 chromosomes, but the number of bins was only 1578. One way to improve bin number is to increase population size, by which more recombination events can be observed. Due to experimental and economic limitations, this option may not be applicable in all cases. Integrated maps provide an effective alternative with which to improve bin density, as markers that cannot be distinguished on some maps may have unique positions on other maps (Allen et al. 2017). However, our study failed to show that this is always the case. The integrated map reported in this study was built from three maps of 8PL, 4PL1 and 4PL2, which contained only the markers retained after binning (Table 2).

We also tried to construct the integrated map using all markers before binning, i.e., 13,254, 10,825 and 13,479 SNPs in 8PL, 4PL1 and 4PL2. The results showed that the inclusion of all useful markers before binning did not always improve the bin density of the integrated map. For example, for chromosome 12 in the integrated map, the bin number was 674 when using markers before binning, which was smaller than that (677) when using markers after binning. The average bin distance was 0.29 cM, which was larger than that (0.25) observed when using markers after binning.

### Comparison of HD and PH QTLs Detected by ICIM and GWAS from the Three Populations

ICIM detected 3, 7 and 9 QTLs for HD and 6, 9 and 10 QTLs for PH in 8PL, 4PL1 and 4PL2 using the three individual maps, respectively. It was not easy to compare the positions of the detected QTLs between maps because of inconsistent marker orders and positions. However, some QTLs had similar physical positions. For example, assuming the length of the confidence interval was 0.5 Mb, the gene *Hd3a* was detected in both 8PL and 4PL1; *Ghd8* was detected in both 8PL and 4PL2; and *sd1* was detected in both 8PL and 4PL1 populations.

In a previous study, Shen et al. (2017) applied GWAS in 8PL and detected two QTLs affecting HD and three affecting PH. ICIM detected all these QTLs detected by the GWAS, i.e., *qHD6.2*, *qHD8*, *qPH1.1*, *qPH1.2* and *qPH12*. In addition, ICIM detected one other QTL affecting HD on chromosome 6 and three other QTLs affecting PH on chromosomes 2, 8 and 11, whose PVE were equal to 5.15, 2.13, 1.42 and 1.28%, respectively. In 4PL1, ICIM also detected all QTLs that were detected by the GWAS, i.e., *qHD6* and *qPH1*. In addition, six other QTLs affecting HD and eight other QTLs affecting PH were identified on various chromosomes. Largest and smallest PVE of these QTL were 11.08 and 1.69%, and average PVE was 3.62%. In 4PL2, two HD and two PH QTLs were identified by the GWAS, one of which was coincident with those identified by ICIM, i.e., *qHD8*. ICIM detected eight other HD QTLs and ten other PH QTLs. Largest and smallest PVE of these QTL were 34.85 and 0.41%, and average PVE was 4.06%. In summary, GWAS missed some strong signals as well as some weak signals in QTL mapping. The reason may be that GWAS is lack of background control leading to a larger sampling error. The selection of GWAS methods also affects the accuracy of QTL detection. ICIM detected more QTLs, and the detected QTLs explained more phenotypic variance than those detected by GWAS, indicating ICIM has more power in QTL detection than GWAS analysis.

For the six cloned genes for HD (i.e. *Hd3a*, *Ghd8* and *HD6*) and PH (*Psd1*, *sd1* and *d61*), both GWAS and ICIM detected them for nine times (Additional file 7: Table S7). *Hd3a* and *sd1* was detected by GWAS in 4PL2, but not found by ICIM; *HD6* and *d61* were detected by ICIM in 4PL2, but not found by GWAS. For the seven times of common genes detected by both methods, the distance of significant SNPs to the gene was smaller by GWAS for five times, and smaller by ICIM for two times. But for most times, the difference between the two methods was small. The reason may be that the most significant SNPs to the gene were deleted by quality control before linkage map construction and QTL mapping. For example, the most significant SNP marker associated with *Psd1* in GWAS was not existed in linkage mapping for 8PL.

### Comparison of QTLs Detected by Meta-Analysis and QTL Mapping from Integrated Map

Meta-analysis was used to project QTLs detected in the three populations onto the integrated map. In total, 19 MQTLs affecting HD and 25 MQTLs affecting PH were detected by meta-analysis. Some QTLs with similar physical positions across populations were still projected to similar linkage positions on the integrated map. For example, both *qPH1.1* in 8PL and *qPH1* in 4PL1 were close to the gene *sd1* on the rice physical map, corresponding to *MqPH1.7* and *MqPH1.6* on the integrated map, respectively. The distance between *MqPH1.7* and *MqPH1.6* was 1.95 cM. Similar results were observed for *qHD8* in 8PL and *qHD8* in 4PL2. However, some exceptions were detected. For example, both *qHD6.2* in 8PL and *qHD6* in 4PL1 were close to the gene *Hd3a* on the physical map, corresponding to *MqHD6.2* and *MqHD6.4* on the integrated map, respectively. The distance between *MqHD6.2* and *MqHD6.4* was 31.24 cM. This phenomenon may have been caused by the difference in marker order between linkage and physical maps.

QTL mapping of the three populations based on an integrated map revealed a total of 24 IQTLs affecting HD and 25 IQTLs affecting PH. Compared with the results from the meta-analysis, the numbers of QTLs were similar. Assuming that the length of the confidence interval was 10 cM, 11 QTLs were coincident between MQTLs and IQTLs. For example, *MqHD6.1* was located at 10.17 cM on chromosome 6, and *IqHD6.1* in 8PL was located at 8.10 cM on the same chromosome. The distance between the two QTLs was 2.07 cM. Whether MQTLs or IQTLs are more reliable requires further investigation. Currently, we are developing a combined analysis in which QTL mapping will be conducted by all individuals from each population and a common genetic map constructed using the combined population.

### Comparison of Genetic Analysis Using Populations Derived from Different Number of Parents

In this study, the number of QTLs detected in 8PL was lower than that in 4PL1 and 4PL2, along with lower PVE. The reason may be as follows. Degree of freedom of the LOD test statistic in 8PL is higher than that in 4PL. LOD threshold from permutation tests in 8PL is always higher than that in 4PL. Some QTLs detected in 4PL may not reach the threshold level in 8PL, and therefore were not reported when 8PL was used. This may explain why more QTL were detected in 4PL in this study. However, when four parents carry same alleles, this locus cannot be detected in the pure-line population derived from the four parents. When other four parents carry different alleles, this locus could be detected in the 8PL population. In this situation, more QTL may be detected in 8PL.Therefore, it is more likely a special case that fewer QTLs were detected in 8PL in this study.

Genetic variance at one locus depends on both allele frequencies and genotypic values. PVE is the proportion of genetic variance to phenotypic variance. PVE in 8PL could be higher or lower than that in 4PL in terms of the same locus. For example, given that there are only two genotypic values at one locus, i.e., 10 and 8, and each genotype have equal frequency. In 4PL, if three genotypic values are 10 and one is 8, the genetic variance is equal to 0.75. For the same locus in 8PL, if four genotypic values are 10 and the other four are 8, the genetic variance is equal to 1, which is higher than that in 4PL. If seven genotypic values are 10 and the other one is 8, the genetic variance is equal to 0.4375, which is lower than that in 4PL. In addition, background genetic variance and random error variance could also be different between 4PL and 8PL. Therefore, it is more likely a special case that lower PVE was detected in 8PL in this study. We understand that further studies are needed to make perspective comparison on QTL detection using populations derived from different number of parents.

From the mapping results, it seems that each parent has unique allele and effect in multi-parent populations. In fact, some parents may share same alleles, i.e. the number of unique effect value may be less than the number of parents. However, due to sampling error, the estimated genotypic values are seldom the same even for same alleles; the estimated genotypic values could be similar for genetically different alleles. Therefore, we doubt the exact number of alleles in 4PL or 8PL can be well determined statistically. For convenience, our QTL mapping method assumes different genotypic values for different parents. Despite this, similar effects among parents were observed in many QTLs, which may represent same alleles, for example in Table 3, *a*_2_ and *a*_3_ of *qHD6.1*, *a*_1_ and *a*_2_ of *qHD6.2*, *a*_4_ and *a*_5_ of *qHD8* and so on. On the other hand, even if we acquired similar estimated effects, it is still hard to determine whether they are the same alleles or not. Instead, biological information is needed to further determine the number of alleles at each QTL, such as DNA sequence and functional analysis.

Compared with bi-parent populations, MAGIC populations contain more alleles and greater genetic diversity. Genetic loci that have no variations in bi-parent populations may have variation in multiple-parent populations because of more alleles are involved. More parents will increase the number of recombination events and improve mapping precision (Huang et al. 2012), which can facilitate QTL detection. However, multiple-parent population is more difficult to develop, because of huge time and labor consumption. More generation of selfing is needed to generate the final pure lines as well. In addition, larger numbers of alleles and marker types at each locus in MAGIC populations complicate the recombination frequency estimation and linkage map construction. Further studies are needed to compare QTL detection using populations from different number of parents.

## Conclusions

In summary, individual linkage maps were constructed from three multi-parent rice populations, and an integrated map was built based on the three individual maps. This study produced the first linkage map of an eight-parent RIL population and the first integrated map constructed from multi-parent populations in rice. These maps are necessary for efficient QTL linkage mapping of quantitatively inherited traits. The integrated map supported the comparison and integration of QTLs detected in individual population. QTLs for the traits HD and PH obtained using ICIM based on the three linkage maps were largely coincident with the QTLs detected by a previous GWAS. In addition, ICIM detected more QTLs and explained more phenotypic variance than did the GWAS. The HD and PH QTLs detected by meta-analysis and ICIM based on the integrated map partly overlapped.

## Supplementary information

**Additional file 1: Table S1.** Phenotypic mean of the eight parents

**Additional file 2: Table S2.** Information on the individual mapping population data used for linkage maps and QTL analysis

**Additional file 3: Table S3.** QTLs for heading date and plant height in the 4PL1 population based on the linkage map

**Additional file 4: Table S4.** QTLs for heading date and plant height in the 4PL2 population based on the linkage map

**Additional file 5: Table S5.** QTLs for heading date and plant height in the 4PL1 population based on the integrated map

**Additional file 6: Table S6.** QTLs for heading date and plant height in the 4PL2 population based on the integrated map

**Additional file 7: Table S7.** Physical position of significant SNPs associated with cloned genes detected by GWAS and ICIM

**Additional file 8: Figure S1.** General information on the markers in the three multi-parent populations

**Additional file 9: Figure S2.** Projection of QTLs detected in the three multi-parent populations on the integrated map

**Additional file 10: Figure S3.** LOD scores across the genome from QTL mapping based on the integrated map

## Data Availability

The authors affirm that all data necessary for confirming the conclusions of this manuscript are represented fully within the manuscript and its tables and figures. Data files for the populations are available from the following website: http://www.isbreeding.net/8-waycross/.

## Abbreviations

4PL1: The first four-parent RIL population
4PL2: The second four-parent RIL population
8PL: Eight-parent RIL population
GAPL: Integrated genetic analysis software for multi-parent pure-line populations
GWAS: Genome-wide association study
HD: Heading date
ICIM: Inclusive composite interval mapping
IQTLs: The QTLs detected based on the integrated map
MAGIC population: Multi-parent advanced generation inter-cross population
MQTLs: Meta-QTLs
PH: Plant height
PLM: Map construction in multi-parent derived pure-line populations
PLQ: QTL or gene detection in multi-parent derived pure-line populations
QTL: Quantitative trait locus; SNP: Single nucleotide polymorphism
