# Supplementary material for: Construction and integration of genetic linkage maps from three multi-parent advanced generation inter-cross populations in rice
Source: Rice (N Y). 2020 Feb 14;13:13. doi: 10.1186/s12284-020-0373-z (PMC7021868; doi:10.1186/s12284-020-0373-z)
Supplement: Supplementary file 1 — Additional file 1: Table S1. Phenotypic mean of the eight parents [file 12284_2020_373_MOESM1_ESM.docx]

**Additional file 1: Table S1.** Phenotypic mean of the eight parents

| Trait | Parent | | | | | | | |
| --- | --- | --- | --- | --- | --- | --- | --- | --- |
|  | A | B | C | D | E | F | G | H |
| HD (d) | 80 | 100 | 89 | 97 | 73 | 76 | 95 | 82 |
|  |  |  |  |  |  |  |  |  |
|  |  |  |  |  |  |  |  |  |
| PH (cm) | 108 | 119.2 | 138.6 | 108.6 | 121.1 | 146.1 | 118.9 | 115.5 |
|  |  |  |  |  |  |  |  |  |
|  |  |  |  |  |  |  |  |  |
