# Supplementary material for: Construction and integration of genetic linkage maps from three multi-parent advanced generation inter-cross populations in rice
Source: Rice (N Y). 2020 Feb 14;13:13. doi: 10.1186/s12284-020-0373-z (PMC7021868; doi:10.1186/s12284-020-0373-z)
Supplement: Supplementary file 2 — Additional file 2: Table S2. Information on the individual mapping population data used for linkage maps and QTL analysis [file 12284_2020_373_MOESM2_ESM.docx]

**Additional file 2: Table S2.** Phenotypic information on the individual mapping populations for linkage maps and QTL analysis

| Trait | Population | Mean | SD | Range | Kurtosis | Skewness | H^2^ (%) |
| --- | --- | --- | --- | --- | --- | --- | --- |
|  |  |  |  |  |  |  |  |
| HD | 8PL | 83.4 | 9.15 | 67.0 - 109.0 | -0.74 | 0.4 | 98.67 |
|  | 4PL1 | 82.2 | 9.61 | 67.0 - 107.0 | -0.71 | 0.47 | 93.46 |
|  | 4PL2 | 81.2 | 8.4 | 66.0 - 108.0 | -0.41 | 0.41 | 97.36 |
| PH | 8PL | 116.88 | 13.44 | 87.7 - 158.6 | -0.12 | 0.46 | 77.66 |
|  | 4PL1 | 119.04 | 17.74 | 89.3 - 170.4 | -0.34 | 0.61 | 77.11 |
|  | 4PL2 | 114.89 | 12.59 | 82.4 - 178.5 | 4.43 | 1.17 | 92.17 |
