# Supplementary material for: Construction and integration of genetic linkage maps from three multi-parent advanced generation inter-cross populations in rice
Source: Rice (N Y). 2020 Feb 14;13:13. doi: 10.1186/s12284-020-0373-z (PMC7021868; doi:10.1186/s12284-020-0373-z)
Supplement: Supplementary file 3 — Additional file 3: Table S3. QTLs for heading date and plant height in the 4PL1 population based on the linkage map [file 12284_2020_373_MOESM3_ESM.docx]

**Additional file 3: Table S3.** QTLs for heading date and plant height in the 4PL1 population based on the linkage map

| QTL | Chr. | Pos. (CI)*^a^* (cM) | Left marker | Right marker | LOD | PVE (%)*^b^* | Genotypic effect | | | | Cloned gene |
| --- | --- | --- | --- | --- | --- | --- | --- | --- | --- | --- | --- |
|  |  |  |  |  |  |  | *a*_1_ | *a*_2_ | *a*_3_ | *a*_4_ |  |
| *qHD3* | 3 | 169.5 (166.85-172.55) | Chr3-5789725 | Chr3-4216773 | 6.97 | 3.93 | 2.04 | -1.40 | 1.67 | -2.31 |  |
| *qHD4* | 4 | 55.2 (55.15-55.95) | Chr4-19240518 | Chr4-18746958 | 7.33 | 2.81 | 3.22 | -1.00 | -1.03 | -1.19 |  |
| *qHD6* | 6 | 63.4 (62.95-64.05) | Chr6-2849460 | Chr6-5144563 | 51.86 | 28.63 | -0.21 | 0.00 | -6.75 | 6.96 | *Hd3a* |
| *qHD7* | 7 | 60.9 (60.45-60.95) | Chr7-5707522 | Chr7-14733912 | 11.02 | 4.16 | -0.34 | -5.23 | 2.31 | 3.26 |  |
| *qHD8.1* | 8 | 23.4 (23.15-24.15) | Chr8-6014968 | Chr8-6006621 | 9.99 | 3.87 | 1.07 | -2.30 | 3.35 | -2.12 |  |
| *qHD8.2* | 8 | 107.2 (106.85-107.55) | Chr8-3847667 | Chr8-3886377 | 25.93 | 11.08 | -4.77 | 3.63 | 5.35 | -4.21 |  |
| *qHD11* | 11 | 127.7 (106.85-107.55) | Chr11-2731894 | Chr11-2963677 | 12.96 | 5.70 | -2.83 | 3.28 | 2.12 | -2.56 |  |
| *qPH1* | 1 | 181.7 (181.25-182.35) | Chr1-38157453 | Chr1-38103681 | 98.95 | 55.80 | -6.98 | -3.00 | 18.73 | -8.76 | *sd1* |
| *qPH3* | 3 | 48.8 (48.65-49.65) | Chr3-1332962 | Chr3-1481616 | 10.35 | 3.00 | 3.08 | 2.37 | -1.93 | -3.52 |  |
| *qPH5.1* | 5 | 70.9 (70.05-71.55) | Chr5-26486089 | Chr5-6359343 | 6.01 | 1.87 | -0.82 | 7.27 | -3.23 | -3.22 |  |
| *qPH5.2* | 5 | 120.9 (120.65-121.15) | Chr5-26384280 | Chr5-26862952 | 6.33 | 1.76 | -3.53 | -1.27 | 2.11 | 2.69 |  |
| *qPH6* | 6 | 146.5 (145.15-146.65) | Chr6-26003396 | Chr6-25756440 | 14.36 | 4.43 | -3.10 | 4.13 | -4.19 | 3.16 |  |
| *qPH7* | 7 | 48.2 (46.85-51.05) | Chr7-930267 | Chr7-774531 | 5.94 | 1.69 | -2.07 | 1.22 | 2.56 | -1.72 |  |
| *qPH8.1* | 8 | 107.1 (106.85-107.45) | Chr8-18414836 | Chr8-3847667 | 7.89 | 2.21 | -3.11 | 2.59 | 4.47 | -3.95 |  |
| *qPH8.2* | 8 | 118.8 (118.65-119.55) | Chr8-9770678 | Chr8-9770496 | 6.83 | 1.91 | -2.17 | 2.59 | 2.18 | -2.60 |  |
| *qPH10* | 10 | 25.5 (24.75-26.05) | Chr10-22707065 | Chr10-22752096 | 6.01 | 2.32 | 3.23 | -2.22 | 1.66 | -2.67 |  |

*^a^* Position in cM and 1-LOD confidence interval (CI)

*^b^* Percentage of phenotypic variance explained
