# Supplementary material for: Construction and integration of genetic linkage maps from three multi-parent advanced generation inter-cross populations in rice
Source: Rice (N Y). 2020 Feb 14;13:13. doi: 10.1186/s12284-020-0373-z (PMC7021868; doi:10.1186/s12284-020-0373-z)
Supplement: Supplementary file 4 — Additional file 4: Table S4. QTLs for heading date and plant height in the 4PL2 population based on the linkage map [file 12284_2020_373_MOESM4_ESM.docx]

**Additional file 4: Table S4.** QTLs for heading date and plant height in the 4PL2 population based on the linkage map

| QTL | Chr. | Pos. (CI) (cM)^a^ | Left marker | Right marker | LOD | PVE (%)*^b^* | Genotypic effect | | | | Cloned gene |
| --- | --- | --- | --- | --- | --- | --- | --- | --- | --- | --- | --- |
|  |  |  |  |  |  |  | *a*_1_ | *a*_2_ | *a*_3_ | *a*_4_ |  |
| *qHD1.1* | 1 | 2.30 (2.15-2.35) | Chr1-30333381 | Chr1-18673799 | 5.94 | 4.26 | -3.21 | -2.86 | -3.62 | 9.68 |  |
| *qHD1.2* | 1 | 31.80 (31.35-32.45) | Chr1-24087706 | Chr1-24575633 | 9.19 | 5.73 | -1.53 | -4.89 | -4.94 | 11.36 |  |
| *qHD2* | 2 | 103.30 (103.15-103.45) | Chr2-16998754 | Chr2-16678368 | 8.20 | 2.69 | -0.47 | -3.17 | 7.13 | -3.49 |  |
| *qHD3.1* | 3 | 13.10 (11.45-13.25) | Chr3-30321470 | Chr3-31928501 | 6.94 | 2.28 | -2.66 | 1.07 | 1.00 | 0.59 | *Hd6* |
| *qHD3.2* | 3 | 105.30 (104.55-106.35) | Chr3-4886779 | Chr3-8369404 | 5.44 | 4.97 | -2.89 | 11.05 | -4.35 | -3.81 |  |
| *qHD6* | 6 | 51.80 (51.55-51.85) | Chr6-1764499 | Chr6-1989861 | 12.11 | 4.11 | -1.44 | -0.58 | 3.09 | -1.07 |  |
| *qHD7* | 7 | 21.70 (21.15-21.95) | Chr7-28375976 | Chr7-25733209 | 7.45 | 2.48 | -1.24 | 2.00 | 0.98 | -1.74 |  |
| *qHD8* | 8 | 125.00 (124.95-125.35) | Chr8-4148475 | Chr8-4155780 | 54.71 | 25.07 | -4.74 | 4.61 | 5.40 | -5.27 | *Ghd8* |
| *qHD11* | 11 | 60.70 (59.95-60.85) | Chr11-2297094 | Chr11-2846415 | 12.18 | 4.24 | -2.22 | 1.05 | -1.41 | 2.58 |  |
| *qPH1.1* | 1 | 124.70 (124.35-124.75) | Chr1-33010552 | Chr1-31811187 | 13.53 | 0.90 | -1.67 | 4.23 | -0.23 | -2.33 |  |
| *qPH1.2* | 1 | 166.70 (177.65-166.95) | Chr1-28136322 | Chr1-28062450 | 164.76 | 34.85 | 17.91 | -17.37 | -19.00 | 18.46 | *d61* |
| *qPH1.3* | 1 | 182.70 (182.65-183.25) | Chr1-41948992 | Chr1-41867990 | 8.05 | 0.51 | 1.84 | 2.88 | -1.74 | -2.98 |  |
| *qPH2* | 2 | 103.90 (103.55-104.15) | Chr2-16678368 | Chr2-16047678 | 8.16 | 0.67 | -2.49 | 1.70 | 3.58 | -2.79 |  |
| *qPH5* | 5 | 11.50 (10.85-12.85) | Chr5-24242729 | Chr5-25619709 | 6.96 | 0.50 | -2.81 | 4.42 | -2.47 | 0.86 |  |
| *qPH7.1* | 7 | 99.90 (99.75-100.35) | Chr7-27677182 | Chr7-26929797 | 19.82 | 1.41 | 3.80 | -4.14 | 4.36 | -4.01 |  |
| *qPH7.2* | 7 | 110.60 (110.55-110.75) | Chr7-16438827 | Chr7-17152825 | 15.43 | 1.43 | -5.06 | -5.10 | 5.09 | 5.08 |  |
| *qPH8* | 8 | 125.80 (125.05-126.45) | Chr8-4155780 | Chr8-3769286 | 13.70 | 1.10 | -2.96 | 5.29 | -0.18 | -2.15 |  |
| *qPH10.1* | 10 | 70.00 (69.85-70.05) | Chr10-9765088 | Chr10-17605232 | 7.51 | 0.55 | -5.07 | 5.85 | -5.96 | 5.19 |  |
| *qPH10.2* | 10 | 78.20 (77.65-79.15) | Chr10-21824431 | Chr10-22722824 | 6.15 | 0.41 | -1.75 | 2.26 | -1.86 | 1.35 |  |

*^a^* Position in cM and 1-LOD confidence interval (CI)

*^b^* Percentage of phenotypic variance explained
