# Supplementary material for: Construction and integration of genetic linkage maps from three multi-parent advanced generation inter-cross populations in rice
Source: Rice (N Y). 2020 Feb 14;13:13. doi: 10.1186/s12284-020-0373-z (PMC7021868; doi:10.1186/s12284-020-0373-z)
Supplement: Supplementary file 5 — Additional file 5: Table S5. QTLs for heading date and plant height in the 4PL1 population based on the integrated map [file 12284_2020_373_MOESM5_ESM.docx]

**Additional file 5: Table S5.** QTLs for heading date and plant height in the 4PL1 population based on the integrated map

| QTL | Chr. | Pos. (CI)*^a^* (cM) | Left marker | Right marker | LOD | PVE (%)*^b^* | Genotypic effect | | | |
| --- | --- | --- | --- | --- | --- | --- | --- | --- | --- | --- |
|  |  |  |  |  |  |  | *a*_1_ | *a*_2_ | *a*_3_ | *a*_4_ |
| *IqHD4* | 4 | 252.41 (251.06-252.46) | Chr4-5826952 | Chr4-5385996 | 6.77 | 1.68 | 2.15 | -2.00 | 2.02 | -2.17 |
| *IqHD5.1* | 5 | 53.50 (53.45-54.55) | Chr5-5613844 | Chr5-5309355 | 10.97 | 10.97 | 2.42 | -1.89 | 1.85 | -2.38 |
| *IqHD5.2* | 5 | 84.60 (84.15-84.95) | Chr5-2399643 | Chr5-3188111 | 10.84 | 2.90 | -2.57 | 2.74 | -2.16 | 1.99 |
| *IqHD6* | 6 | 150.4 (150.15-150.45) | Chr6-30839032 | Chr6-2849460 | 66.50 | 24.88 | -4.11 | -1.82 | -3.86 | 9.79 |
| *IqHD8* | 8 | 17.3 (17.25-17.45) | Chr8-3600980 | Chr8-3625032 | 13.10 | 3.66 | -1.75 | -0.65 | 5.72 | -3.32 |
| *IqHD11.1* | 11 | 2.9 (2.45-3.05) | Chr11-17684697 | Chr11-17118954 | 9.27 | 2.48 | -1.95 | 2.34 | -3.28 | 2.89 |
| *IqHD11.2* | 11 | 86.00 (84.75-86.85) | Chr11-22250199 | Chr11-28942960 | 6.41 | 1.73 | 1.59 | -2.18 | 2.05 | -1.47 |
| *IqHD11.3* | 11 | 203.40 (203.35-203.55) | Chr11-17669949 | Chr11-2208112 | 6.18 | 1.91 | -1.40 | 2.30 | 1.30 | -2.20 |
| *IqHD12* | 12 | 28.30 (27.35-28.95) | Chr12-24123500 | Chr12-24224755 | 5.82 | 1.46 | 1.94 | -2.25 | -1.20 | 1.51 |
| *IqPH1* | 1 | 358.21 (358.16-358.26) | Chr1-38503485 | Chr1-38103681 | 80.17 | 16.73 | -5.02 | -6.96 | 16.97 | -5.00 |
| *IqPH2* | 2 | 128.70 (128.45-129.25) | Chr2-16001552 | Chr2-16678368 | 6.65 | 1.09 | -3.82 | -2.12 | 4.05 | 1.89 |
| *IqPH3* | 3 | 201.20 (199.153-202.25) | Chr3-1220125 | Chr3-1091399 | 12.39 | 1.80 | 4.02 | 4.51 | -3.81 | -4.72 |
| *IqPH4.1* | 4 | 149.90 (149.85-149.95) | Chr4-12587841 | Chr4-12539446 | 13.18 | 1.52 | 7.47 | -2.07 | -3.20 | -2.20 |
| *IqPH4.2* | 4 | 262.91 (262.56-263.16) | Chr4-24468917 | Chr4-24515261 | 6.67 | 0.73 | 3.18 | -3.22 | 1.72 | -1.68 |
| *IqPH5* | 5 | 100.60 (99.85-101.05) | Chr5-29255690 | Chr5-19738561 | 10.63 | 1.37 | -4.72 | 5.30 | 3.20 | -3.78 |
| *IqPH6* | 6 | 9.9 (9.75-10.65) | Chr6-1764499 | Chr6-27043225 | 19.96 | 2.40 | -3.62 | 3.53 | -4.44 | 4.53 |
| *IqPH8* | 8 | 160.60 (157.65-160.85) | Chr8-885572 | Chr8-9018033 | 7.45 | 0.94 | -3.19 | 1.04 | 3.67 | -1.52 |
| *IqPH9* | 9 | 83.70 (83.65-84.75) | Chr9-20802858 | Chr9-20760991 | 8.15 | 0.92 | 2.72 | -2.63 | -2.60 | 2.51 |
| *IqPH12* | 12 | 61.40 (59.65-62.15) | Chr12-23187925 | Chr12-23938101 | 5.87 | 0.66 | 2.80 | 0.85 | -1.33 | -2.31 |

*^a^* Position in cM and 1-LOD confidence interval (CI)

*^b^* Percentage of phenotypic variance explained
