# Supplementary material for: Construction and integration of genetic linkage maps from three multi-parent advanced generation inter-cross populations in rice
Source: Rice (N Y). 2020 Feb 14;13:13. doi: 10.1186/s12284-020-0373-z (PMC7021868; doi:10.1186/s12284-020-0373-z)
Supplement: Supplementary file 6 — Additional file 6: Table S6. QTLs for heading date and plant height in the 4PL2 population based on the integrated map [file 12284_2020_373_MOESM6_ESM.docx]

**Additional file 6: Table S6.** QTLs for heading date and plant height in the 4PL2 population based on the integrated map

| QTL | Chr. | Pos. (CI)*^a^* (cM) | Left marker | Right marker | LOD | PVE (%)*^b^* | Genotypic effect | | | |
| --- | --- | --- | --- | --- | --- | --- | --- | --- | --- | --- |
|  |  |  |  |  |  |  | *a*_1_ | *a*_2_ | *a*_3_ | *a*_4_ |
| *IqHD3* | 3 | 182.20 (181.55-182.35) | Chr3-16728414 | Chr3-26153349 | 13.80 | 7.55 | 3.80 | -4.56 | -2.64 | 3.41 |
| *IqHD5.1* | 5 | 166.30 (165.95-166.55) | Chr5-16960525 | Chr5-16649781 | 7.32 | 4.23 | -1.76 | 1.90 | -1.04 | 0.91 |
| *IqHD5.2* | 5 | 248.41 (247.66-248.56) | Chr5-23787388 | Chr5-23780875 | 8.14 | 4.20 | -2.24 | 1.29 | -0.67 | 1.62 |
| *IqHD6.1* | 6 | 30.70 (30.25-30.75) | Chr6-21397060 | Chr6-21523247 | 6.51 | 3.30 | 1.60 | -1.22 | -1.33 | 0.95 |
| *IqHD6.2* | 6 | 92.80 (92.15-93.05) | Chr6-3305201 | Chr6-2927171 | 18.67 | 10.27 | -2.86 | -3.59 | 6.16 | 0.29 |
| *IqHD7* | 7 | 182.50 (182.35-183.05) | Chr7-29117736 | Chr7-28430850 | 14.11 | 8.43 | -3.12 | 1.51 | 4.84 | -3.23 |
| *IqHD8.1* | 8 | 51.90 (51.65-51.95) | Chr8-4077566 | Chr8-4201278 | 14.98 | 8.44 | -1.93 | 2.57 | 1.72 | -2.36 |
| *IqHD8.2* | 8 | 177.50 (176.35-178.05) | Chr8-3549057 | Chr8-4080922 | 7.50 | 4.50 | -1.69 | -0.28 | 1.85 | 0.12 |
| *IqHD10* | 10 | 56.10 (55.95-56.25) | Chr10-18139875 | Chr10-15817063 | 7.71 | 7.41 | 1.55 | 2.33 | -3.41 | -0.46 |
| *IqHD11* | 11 | 172.40 (171.95-173.75) | Chr11-2759954 | Chr11-1844865 | 6.15 | 4.59 | -3.98 | -0.48 | 5.86 | -1.39 |
| *IqPH1.1* | 1 | 43.30 (42.85-43.95) | Chr1-28062450 | Chr1-28135962 | 88.19 | 12.39 | -10.33 | 10.36 | 11.00 | -11.04 |
| *IqPH1.2* | 1 | 103.90 (103.85-103.95) | Chr1-28684095 | Chr1-28136322 | 119.95 | 21.74 | 13.57 | -14.51 | -12.17 | 13.11 |
| *IqPH1.3* | 1 | 118.00 (117.95-118.25) | Chr1-38207031 | Chr1-36246564 | 10.45 | 0.85 | -3.26 | 5.27 | 1.48 | -3.49 |
| *IqPH1.4* | 1 | 249.71 (249.66-249.86) | Chr1-12519146 | Chr1-19352070 | 7.03 | 0.61 | 2.73 | -2.09 | 3.05 | -3.68 |
| *IqPH1.5* | 1 | 338.91 (338.56-339.46) | Chr1-35658253 | Chr1-35625693 | 7.98 | 0.62 | -1.10 | 5.21 | 0.15 | -4.26 |
| *IqPH2.1* | 2 | 55.30 (52.95-55.85) | Chr2-3568574 | Chr2-16580052 | 8.12 | 0.76 | -1.28 | 3.85 | -4.44 | 1.87 |
| *IqPH2.2* | 2 | 121.10 (120.55-121.25) | Chr2-23858322 | Chr2-21002577 | 6.15 | 0.53 | 2.79 | -2.11 | 1.37 | -2.04 |
| *IqPH3.1* | 3 | 91.90 (94.55-94.95) | Chr3-4216773 | Chr3-14228701 | 9.00 | 0.73 | -2.19 | -4.07 | 3.95 | 2.32 |
| *IqPH3.2* | 3 | 121.30 (120.05-121.25) | Chr3-27709885 | Chr3-33080942 | 7.28 | 0.58 | -3.31 | 1.93 | 2.98 | -1.60 |
| *IqPH7* | 7 | 94.80 (94.55-94.95) | Chr7-2149971 | Chr7-20731067 | 5.90 | 0.66 | -1.65 | -4.26 | -3.47 | 9.37 |
| *IqPH8* | 8 | 51.90 (51.65-52.15) | Chr8-4077566 | Chr8-4201278 | 6.93 | 0.55 | -2.88 | 2.80 | 1.42 | -1.33 |
| *IqPH12* | 12 | 133.60 (133.55-134.25) | Chr12-22830418 | Chr12-23517419 | 20.52 | 1.78 | -5.75 | 6.32 | -6.01 | 5.45 |

*^a^* Position in cM and 1-LOD confidence interval (CI)

*^b^* Percentage of phenotypic variance explained
