# Supplementary material for: Construction and integration of genetic linkage maps from three multi-parent advanced generation inter-cross populations in rice
Source: Rice (N Y). 2020 Feb 14;13:13. doi: 10.1186/s12284-020-0373-z (PMC7021868; doi:10.1186/s12284-020-0373-z)
Supplement: Supplementary file 7 — Additional file 7: Table S7. Physical position of significant SNPs associated with cloned genes detected by GWAS and ICIM [file 12284_2020_373_MOESM7_ESM.docx]

**Additional file 7: Table S7.** Physical position of significant SNPs associated with cloned genes detected by GWAS and ICIM

| Trait | Cloned  gene | Location  (bp) | GWAS | | | ICIM | | | | | | |  |
| --- | --- | --- | --- | --- | --- | --- | --- | --- | --- | --- | --- | --- | --- |
|  |  |  | 4PL1 (bp) | 4PL2 (bp) | 8PL  (bp) | | 4PL1 | | 4PL2 | | 8PL | | |
|  |  |  |  |  |  |  | Left marker  (bp) | Right marker (bp) | Left marker (bp) | Right marker (bp) | Left marker (bp) | Right marker (bp) | |
| HD | *Hd3a* | 2940004 - 2942452 | 2927171 | 2902951 | 2959067 | | 2849460 | 5144563 |  |  | 3305201 | 2927171 | |
|  | *Ghd8* | 4333846 - 4334739 |  | 4265867 | 4265867 | |  |  | 4148475 | 4155780 | 4701356 | 4892161 | |
|  | *HD6* | 31508811 - 31514460 |  |  |  | |  |  | 30321470 | 31928501 |  |  | |
| PH | *Psd1* | 35129858 - 35130917 |  |  | 36724510 | |  |  |  |  | 37101140 | 37595895 | |
|  | *sd1* | 38381423 - 38384165 | 38398521 | 38547762 | 38381991 | | 38157453 | 38103681 |  |  | 38264136 | 38383144 | |
|  | *d61* | 29927543 - 29931487 |  |  |  | |  |  | 28136322 | 28062450 |  |  | |
