# Supplementary material for: Construction and integration of genetic linkage maps from three multi-parent advanced generation inter-cross populations in rice
Source: Rice (N Y). 2020 Feb 14;13:13. doi: 10.1186/s12284-020-0373-z (PMC7021868; doi:10.1186/s12284-020-0373-z)
Supplement: Supplementary file 8 — Additional file 8: Figure S1. General information on the markers in the three multi-parent populations [file 12284_2020_373_MOESM8_ESM.docx]

**Additional file 8: Figure S1.** General information on the markers in the three multi-parent populations. A, Missing rate; B, frequency of heterozygosity; C, number of common markers. In A, 80 and 150 were added to missing rate of markers for 4PL1 and 4PL2, respectively.
