# Supplementary material for: Construction and integration of genetic linkage maps from three multi-parent advanced generation inter-cross populations in rice
Source: Rice (N Y). 2020 Feb 14;13:13. doi: 10.1186/s12284-020-0373-z (PMC7021868; doi:10.1186/s12284-020-0373-z)
Supplement: Supplementary file 9 — Additional file 9: Figure S2. Projection of QTLs detected in the three multi-parent populations on the integrated map [file 12284_2020_373_MOESM9_ESM.docx]

**Additional file 9: Figure S2.** Projection of QTLs detected in the three multi-parent populations on the integrated map
