# Supplementary material for: Construction and integration of genetic linkage maps from three multi-parent advanced generation inter-cross populations in rice
Source: Rice (N Y). 2020 Feb 14;13:13. doi: 10.1186/s12284-020-0373-z (PMC7021868; doi:10.1186/s12284-020-0373-z)
Supplement: Supplementary file 10 — Additional file 10: Figure S3. LOD scores across the genome from QTL mapping based on the integrated map [file 12284_2020_373_MOESM10_ESM.docx]

**Additional file 10: Figure S3.** LOD scores across the genome from QTL mapping based on the integrated map. A, 8PL; B, 4PL1; C, 4PL2. Fifty, seventy and thirty were added to LOD score of plant height in A, B and C, respectively.
